# Enhanced Human Activity Recognition Using Wi-Fi Sensing: Leveraging Phase and Amplitude with Attention Mechanisms

**DOI:** 10.3390/s25041038

**Published:** 2025-02-09

**Authors:** Thai Duy Quy, Chih-Yang Lin, Timothy K. Shih

**Affiliations:** 1 Department of Computer Science and Information Engineering, National Central University, Taoyuan City 320317, Taiwan; quytd@dlu.edu.vn; 2 Department of Mechanical Engineering, National Central University, Taoyuan City 320317, Taiwan

**Keywords:** Wi-Fi sensing, channel state information (CSI), human activity recognition (HAR), phase and amplitude, multi-head attention, multi-scale convolutional neural networks, gate residual network

## Abstract

Wi-Fi-based human activity recognition (HAR) is a non-intrusive and privacy-preserving method that leverages Channel State Information (CSI) for identifying human activities. However, existing approaches often struggle with robust feature extraction, especially in dynamic and multi-environment scenarios, and fail to effectively integrate amplitude and phase features of CSI. This study proposes a novel model, the Phase–Amplitude Channel State Information Network (PA-CSI), to address these challenges. The model introduces two key innovations: (1) a dual-feature approach combining amplitude and phase features for enhanced robustness, and (2) an attention-enhanced feature fusion mechanism incorporating multi-scale convolutional layers and Gated Residual Networks (GRN) to optimize feature extraction. Experimental results demonstrate that the proposed model achieves state-of-the-art performance on three datasets, including StanWiFi (99.9%), MultiEnv (98.0%), and the MINE lab dataset (99.9%). These findings underscore the potential of the PA-CSI model to advance Wi-Fi-based HAR in real-world applications.

## 1. Introduction

Human activity recognition (HAR) [1] is a significant area of research in computer vision and pattern recognition that focuses on identifying and classifying human activities from multimedia environments. HAR can be achieved using various technologies, including camera-based [2], sensor-based [1], and radar-based technologies [3], as well as Wi-Fi [1,4]. Camera-based HAR relies on video data to analyze and classify actions. While it offers high-resolution and detailed visual information, it raises privacy concerns and is sensitive to lighting conditions and occlusions. Sensor-based HAR utilizes wearable devices like accelerometers and gyroscopes to capture movement data, providing accuracy and robustness but requiring user compliance, while also potentially being intrusive [1,5]. Radar-based HAR uses radio waves to detect motion, offering privacy advantages and working well in low-light conditions, but it can be affected by interference and requires complex signal processing. In contrast, Wi-Fi-based HAR leverages the analysis of CSI to recognize actions by detecting changes in signal propagation caused by human movement. This method is advantageous for its non-intrusiveness, cost-effectiveness, and ability to work through walls and in various lighting conditions. However, Wi-Fi-based HAR may face challenges in environments with high signal noise and requires sophisticated algorithms to interpret the data accurately [6,7]. Wi-Fi-based HAR stands out due to its balance of privacy, convenience, and practicality in real-world applications.

**Figure 1:**
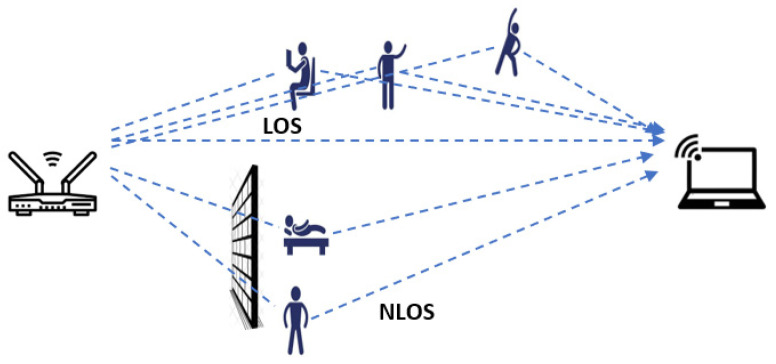
The propagation of Wi-Fi signals from the transmitter to the receiver in LOS/NLOS scenarios.

In Wi-Fi sensing, two critical metrics are commonly used to represent the characteristics of a Wi-Fi signal: the Received Signal Strength Indicator (RSSI) and Channel State Information (CSI) [5,8]. These signals can propagate and synthesize through either line-of-sight (LOS) or non-line-of-sight (NLOS) scenarios (Figure 1). RSSI is a measure of the power level that a receiver detects from a transmitted signal, often used to estimate the distance between the transmitter and receiver. It provides a coarse measure of signal strength but lacks detailed information about the multipath effects and the fine-grained behavior of the signal [5]. On the other hand, CSI offers a more comprehensive representation by capturing the amplitude and phase of the signal across multiple subcarriers within a Wi-Fi channel. This allows CSI to provide detailed insights into the propagation environment, including reflections, scattering, and the impact of obstacles [8]. While RSSI is easier to obtain and requires less computational processing, CSI is preferred in advanced Wi-Fi sensing applications, such as human activity recognition and indoor localization, due to its ability to capture rich spatial and temporal information about the signal’s interactions with the environment.

Channel State Information (CSI) comprises multiple extractable features that reveal characteristics of the wireless environment, such as Time of Flight (ToF), amplitude, phase, Direction of Arrival (DoA), Angle of Arrival (AoA), and phase shift [8]. Each feature provides unique information; for example, ToF enables precise distance estimation, while DoA and AoA are critical for identifying the signal’s spatial direction. In human activity recognition (HAR) applications, amplitude and phase are two particularly important features. Amplitude reflects the magnitude of signal variations, whereas phase provides detailed insights into signal propagation, including phase shifts caused by reflections and scattering. However, Wi-Fi sensing research has largely focused on the amplitude component of CSI, often neglecting phase information in the received signal. Additionally, CSI data are represented as a series of time-varying values across subcarriers. Most recognition models, however, concentrate on the temporal dimension and tend to overlook inter-subcarrier relationships, which could capture variations in body posture or actions, such as standing up and sitting down.

To address these gaps, this study seeks to utilize relevant features in conjunction with attention mechanisms across both temporal and channel dimensions, enhancing the accuracy and robustness of Wi-Fi-based applications for human activity recognition (HAR). In summary, the primary contributions of this paper to Wi-Fi sensing for HAR are as follows:

- We propose an attention-based model that effectively utilizes both phase and amplitude components to improve HAR performance. While prior studies primarily focused on amplitude, our approach incorporates phase data, which provides complementary insights into human activity. By optimizing feature extraction for both amplitude and phase, our method significantly enhances recognition accuracy and robustness across a wide range of activities and environments.
- We introduce an attention-based feature fusion mechanism that integrates spatial and temporal features. This includes the implementation of multi-scale convolutional layers, enabling the network to efficiently capture both local and global patterns. In addition, a Gated Residual Network (GRN) is incorporated into our framework as part of the feature fusion process. GRNs can selectively retain or discard information, improving learning efficiency and reducing unnecessary complexity. This adaptation enhances classification accuracy and addresses the limitations of traditional networks. Our focus lies in how the GRN is specifically adapted and utilized in conjunction with attention mechanisms to maximize the utility of multi-scale features.
- Our model is rigorously evaluated using three datasets, including two publicly available datasets and a custom dataset collected for this study. The results demonstrate that our approach achieves superior accuracy and performance compared to existing state-of-the-art (SOTA) models.

## 2. Literature Review

The Received Signal Strength Indicator (RSSI), a metric used to measure transmission channel power, has been extensively studied and applied across various domains, including human activity recognition (HAR) [9,10,11,12,13,14]. Despite its utility, RSSI’s efficiency is constrained. In contrast, Channel State Information (CSI) offers a more comprehensive and detailed representation, providing a four-dimensional matrix that captures the characteristics of transmission and reception channels, subcarriers, and temporal packets. Due to its complexity, numerous studies have explored the application of CSI in HAR using advanced deep learning techniques, such as Convolutional Neural Networks (CNNs), Long Short-Term Memory (LSTM) networks, and Transformer models.

As interest in Wi-Fi CSI methods continues to grow, numerous datasets containing CSI data have been developed for diverse applications. These datasets have become foundational for many researchers, serving as the basis for experimental studies documented in a wide range of published literature. Table 1 provides an overview of commonly used public datasets and their descriptions, facilitating an understanding of their utility and scope.

### 2.1. Wi-Fi CSI Based on CNN and LSTM Approaches

The intricate features of CSI data, including phase and amplitude, have been effectively utilized in CNN models, yielding significant advancements. Wang et al. [21] introduced a CSI-based human activity recognition and monitoring system (CARM), a CSI-based method for HAR and monitoring. This model integrated two modules: the CSI-speed module, which captured the relationship between CSI dynamics and human motion, and the CSI-activity module, which correlated movement speed with activity. By leveraging the phase component of CSI, the model was tested on their dataset and achieved 96% accuracy in HAR while demonstrating robustness across different environments. Although the method was accurate, cost-effective, and privacy-preserving when using standard Wi-Fi devices, it was sensitive to noise, impacted Wi-Fi performance, and required extensive training.

Similarly, Alsaify et al. [15] proposed the MultiEnv dataset, evaluating system performance in office and hall environments under line-of-sight (LOS) and non-line-of-sight (NLOS) conditions. Their robust five-stage preprocessing and feature extraction pipeline achieved 91.27% accuracy on their dataset [15,22]. However, environmental factors such as distance and interference, as well as similar movement patterns (e.g., falling versus sitting), led to higher misclassification rates.

While CNN-based models demonstrated promising results, they lacked the ability to exploit the temporal characteristics of CSI data. Consequently, temporal-based approaches utilizing LSTM networks have gained attention. For example, Chen et al. [23] developed the ABLSTM model (attention-based bidirectional LSTM), which was designed to extract representative temporal features from sequential CSI data in both directions. The ABLSTM framework achieved state-of-the-art accuracy (≥95%) in Wi-Fi CSI-based HAR, evaluated on the StanWiFi dataset and their own dataset, by integrating bidirectional LSTM and attention mechanisms. However, this model required significant computational resources, faced challenges in cross-environment scenarios, and did not address multi-user activity recognition.

Yadav et al. [24] proposed CSITime, an enhanced version of the InceptionTime network, which achieved state-of-the-art accuracies across three public datasets: 98.2% on ARIL, 98.88% on StanWiFi, and 99.09% on SignFi. CSITime incorporated data augmentation and advanced optimizations within a streamlined InceptionTime-based architecture. However, the model faced challenges in high-interference environments and multi-user scenarios, while demanding high computational resources.

To address spatial and temporal aspects of CSI data, several studies combined CNN and LSTM architectures. For instance, Moshiri et al. [25] employed Raspberry Pi devices to extract amplitude CSI signals for seven activities, converting them into 2D images using pseudo-color plots. Among four evaluated models (1D-CNN, 2D-CNN, LSTM, and bidirectional LSTM), the 2D-CNN achieved 95% accuracy on their collected data. Similarly, Salehinejad and Valaee [26] introduced LiteHAR, which uses randomly initialized convolution kernels for feature extraction, achieving 93% accuracy on the StanWiFi dataset. Shalaby et al. [27] compared deep learning models on the StanWiFi dataset, with a CNN-GRU model achieving 99.31% accuracy and its attention-based variant reaching 99.16%. Recently, Islam et al. [28] proposed the STC-NLSTMNet model (spatio-temporal convolution with nested long short-term memory), which integrated spatial–temporal convolution and nested LSTMs, achieved 99.88% and 98.20% accuracy on public datasets (StanWiFi and MultiEnv). This model was a robust and efficient method for HAR, achieving SOTA performance by integrating spatial and temporal features. However, the model had limitations in NLOS environments and multi-user settings.

### 2.2. Attention-Based Approaches

Traditional models such as CNNs and LSTMs were limited in capturing interdependencies within the same data dimension. In recent years, Transformer models have gained prominence due to their multi-head attention mechanism [29]. Researchers have applied these models to Wi-Fi-based HAR systems with notable success.

Ding et al. (2022) introduced a Channel–Time–Subcarrier Attention Mechanism (CTS-AM) to enhance location-independent HAR. The model CTS-AM demonstrated innovation in feature extraction, resource efficiency, and flexibility, outperforming existing methods like CNN and Wi-Hand. However, the system was limited to single-actor scenarios, assumed consistent motion during data collection, and recognized only predefined activities. This model achieved more than 90% average accuracy on their dataset across various locations with limited training samples [30]. Yang et al. (2023) [31] proposed WiTransformer, which adapted two Transformer architectures—the United Spatiotemporal Transformer (UST) and the Separated Spatiotemporal Transformer (SST)—to improve recognition accuracy and robustness in complex environments [31]. The UST integrated spatial and temporal features through early fusion in a single transformer encoder, resulting in high accuracy, computational efficiency, and robustness across tasks of varying complexity. In contrast, the SST processed spatial and temporal features separately using two transformer encoders, offering flexibility, but at the cost of increased complexity and resource demands. The SST struggled with generalization on smaller datasets, exhibited sensitivity to imbalanced data, and lacked the inductive biases needed for effective cross-domain adaptation. The UST’s efficient and unified approach made it more practical and robust compared to the SST, particularly for real-world applications. They experimented with two models on the Widar 3.0 dataset and achieved an overall recognition accuracy of 86.16%.

Some studies also explored two-way relationships within CSI data. Li et al. (2021) [32] developed the Two-stream Convolution Augmented Human Activity Transformer (THAT) model, which uses a dual convolution-augmented HAR layer to capture channel and temporal structures. This model effectively accommodated variations in activity speed and blank intervals using Gaussian encoding. Yang et al. (2023) [19] applied Vision Transformers (ViT) to Wi-Fi sensing tasks, leveraging spatial and temporal features to handle complex data relationships. However, ViT models required substantial training data for optimal performance.

Although a few studies have utilized phase information for HAR, the majority of existing works predominantly focus on amplitude features, leaving phase information underexplored or insufficiently integrated with amplitude. While existing attention-based methods primarily focused on either spatial or temporal dimensions, our proposed PA-CSI model incorporates attention across both. By leveraging multi-head attention mechanisms and multi-scale convolutional neural networks, the PA-CSI model effectively integrates amplitude and phase features to achieve superior performance. Moreover, the use of Gaussian Range Encoding (GRE) ensures robust temporal feature representation, addressing limitations observed in CTS-AM and THAT models.

## 3. Materials and Methods

### 3.1. Channel State Information (CSI)

Channel State Information (CSI) refers to the detailed properties of a communication channel in wireless communication systems. CSI encompasses information about the channel’s characteristics, including path loss, fading, delay spread, and interference [33]. CSI is typically obtained through channel estimation techniques, which involve measuring the channel response to known pilot signals transmitted between the sender and receiver. Mathematically, CSI can be represented by the channel matrix H, which characterizes the effect of the channel on the transmitted signal. If *x* is the transmitted signal and *y* is the received signal, *N* is the total number of subcarriers, and η represents the noise in the system, the relationship between *x* and *y* for each *i-th* subcarrier can be expressed as follows (1):

(1) $$y_{i}=H_{i}x_{i}+\eta ,\;i=1,\;2,\ldots ,N$$

The matrix $H_{i}$ presents the relations from transmitter (*T*) and receiver (*R*) as (2):

(2) $$H_{i}={\left[\begin{array}{ccc} h_{11} & \cdots & h_{1R}\\ \vdots & \ddots & \vdots \\ h_{1T} & \cdots & h_{TR} \end{array}\right]}_{i},$$

where each $h_{rt}$ contains complex values, represented as shown in (3):

(3) $$h_{rt}=ℜ\left(h_{rt}\right)+ℑ\left(h_{rt}\right)j,\;j^{2}=-1$$

Each *h_rt_* value consists of both the real $ℜ\left(h_{rt}\right)$ and imaginary $ℑ\left(h_{rt}\right)$ parts, which encapsulate the amplitude and phase changes imposed by the channel. The amplitude ($A_{rt})$ and phase ($\phi_{rt})$ are calculated as given in Equations (4) and (5):

(4) $$A_{rt}=\sqrt{{\left(ℜ\left(h_{rt}\right)\right)}^{2}+{\left(ℑ\left(h_{rt}\right)\right)}^{2}},$$

(5) $$\phi_{rt}=arctan\left(\frac{ℑ\left(h_{rt}\right)}{ℜ\left(h_{rt}\right)}\right),$$

**Figure 2:**
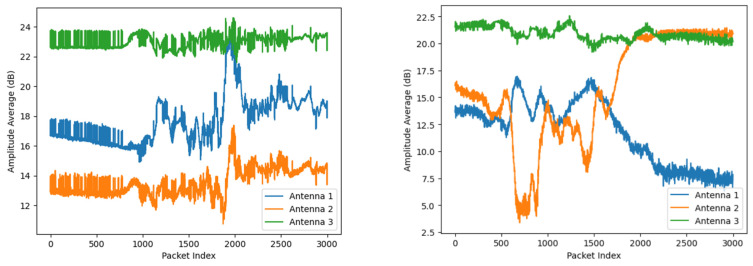

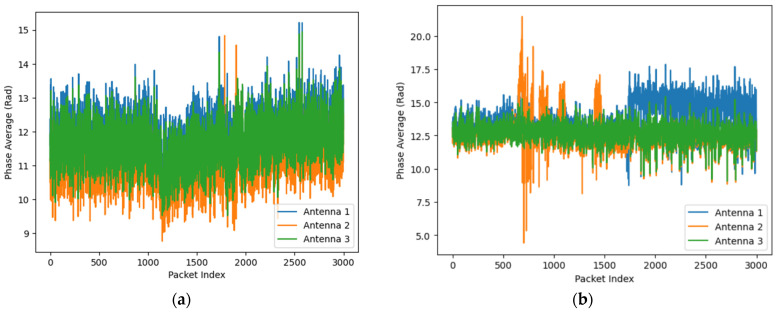
Visualization of Wi-Fi CSI amplitude (above) and phase (below). (**a**) Signal description of the falling action across 3 antennas; (**b**) Signal description of the sitting down action across 3 antennas.

An example visualization of falling and sitting down activities, represented by the averaged amplitude and phase values of Wi-Fi Channel State Information (CSI), is shown in Figure 2.

### 3.2. Datasets

In our experiment, we selected and evaluated three datasets: MultiEnv (three environments) [15], StanWiFi [16], and a dataset collected by our research team. The rationale for selecting these datasets was their inclusion of both CSI phase and amplitude values, which are critical for the model’s ability to effectively utilize these inputs.

#### 3.2.1. StanWiFi

The StanWiFi dataset was collected by Yousefi et al. (2017) [16] in a line-of-sight (LOS) environment. The setup included a single Wi-Fi router with one antenna as the transmitter and three antennas as the receiver, installed on a laptop equipped with an Intel^®^ 5300 NIC. The transmitter and receiver were three meters apart. Each session, recorded at a sampling rate of 1000 Hz, lasted 20 s. This dataset consists of six activities—fall, run, lie down, walk, sit down, and stand up—performed by six participants, each repeating them 20 times. In total, the dataset comprises 577 samples.

#### 3.2.2. Multiple Environment (MultiEnv)

The MultiEnv dataset was collected by Alsaify et al. (2020) [15] across three indoor environments: an office and a hall for line-of-sight (LOS) scenarios, and a non-line-of-sight (NLOS) scenario with a wooden barrier separating the transmitter and receiver. The distance between the transmitter and receiver was set at 3.7 m, with an 8-centimeter wall as the barrier. Data were collected using two computers: one serving as a single-antenna transmitter and the other as a three-antenna receiver, both equipped with an Intel^®^ 5300 NIC. As listed in Table 2, the dataset includes six activity classes, with a total of 3000 samples (30 subjects × 5 experiments × 20 trials).

#### 3.2.3. Our Research Team Dataset (MINE Lab Dataset)

**Figure 3:**
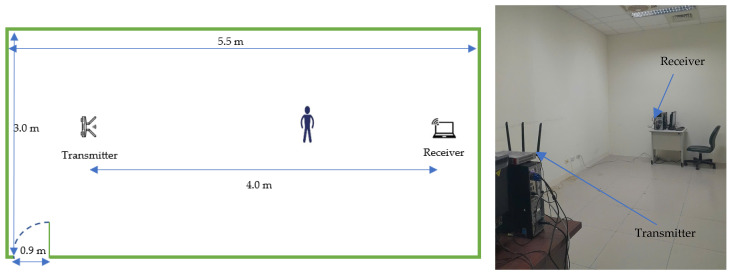
MINE lab environment.

This dataset was collected in an office (LOS) within our laboratory (5.5 × 3 m). The setup utilized Intel^®^ 5300 NIC devices, each equipped with three antennas for both the transmitter and receiver, placed 4.0 m apart (Figure 3). The experiment involved five participants performing six activities: standing up and squatting, raising and lowering the right hand, opening and closing the arms, kicking the right and the left leg. In total, the dataset comprises 216 collected samples.

### 3.3. Methods

**Figure 4:**
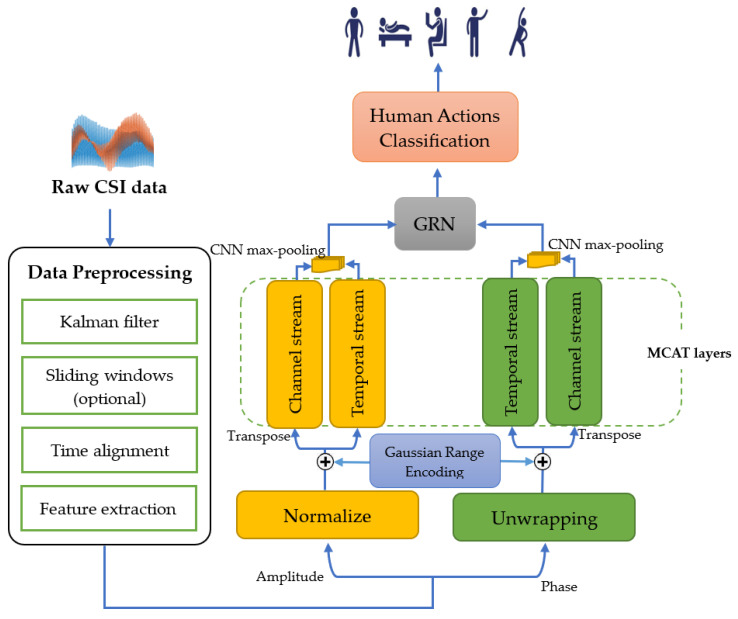
The architecture of the proposed HAR system (CA-CSI).

Figure 4 illustrates a pipeline of the proposed HAR system in this paper. It begins with raw CSI data, which undergoes preprocessing steps including Kalman filtering [34], sliding windows (optional), time alignment, and feature extraction. After preprocessing, the CSI data are extracted into two components: amplitude and phase. The amplitude data are normalized, while the phase data are unwrapped, and both features are passed through four Multi-scale Convolution Augmented Transformer (MCAT) layers [33]. Each MCAT layer processes data along two streams: the data are kept in their original format for the temporal stream, while it is transposed to feed into the channel stream. Gaussian Range Encoding is applied to preserve temporal order. The outputs of the MCAT layers are combined using a CNN with max-pooling layers. The combined outputs are then subsequently fed into a Gated Residual Network (GRN) [35,36], which includes dense layers with exponential linear unit (ELU) activations, layer normalization, and a final sigmoid activation. The GRN integrates the processed amplitude and phase features to classify human actions. This system effectively enables the classification of human activities from CSI Wi-Fi signals as they interact with their environment.

#### 3.3.1. Preprocessing: Kalman Filter

The Kalman filter is a recursive algorithm that efficiently estimates the state of a dynamic system from a series of noisy measurements [34], to minimize *η*, as described in Equation (1). It operates in two main phases: prediction and update. During the prediction phase, the filter uses the system’s previous state and a mathematical model to predict the next state, while also estimating the associated uncertainty. In the update phase, it updates the estimate by incorporating new measurements and reducing uncertainty based on the discrepancy between the predicted and observed data. The Kalman filter assumes that the errors in both the system and the measurements are normally distributed, following a Gaussian distribution, making it optimal for linear systems affected by Gaussian noise.

#### 3.3.2. Preprocessing: Sliding Windows

**Figure 5:**
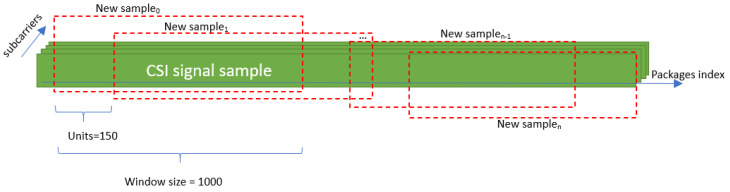
Sliding window applied to the StanWiFi dataset and our dataset.

In this phase, appropriate preprocessing techniques are applied to the collected CSI data depending on the characteristics of each dataset. For both the StanWiFi dataset and our own dataset, we use a sliding window approach to generate additional samples, which is also essential for real-time action prediction when the exact point at which the action will occur within the signal is unknown (Figure 5). Specifically, each instance is shifted by 150, and a window size of 1000 is used for each sample. As a result, the data augmentation process leads to an expansion of the StanWiFi dataset from 577 records to 3119 records, while our dataset increases from 216 samples to 2111 samples.

#### 3.3.3. Preprocessing: Time Alignment

**Figure 6:**
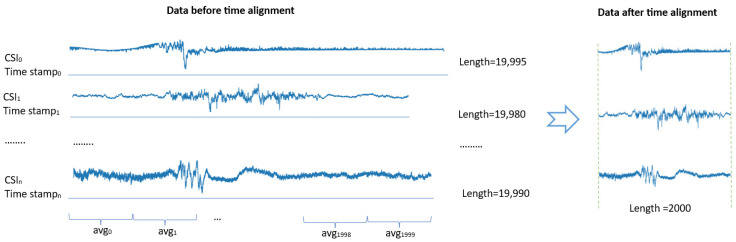
An illustrate of time alignment on three samples in the StanWiFi dataset.

In the datasets, CSI signal sample lengths vary, and the signals exhibit high density with similar characteristics. To address this issue, we propose a time alignment algorithm designed to standardize the sample sizes using collection times and average signal values. This algorithm processes CSI data based on timestamps, normalizing them to a specified maximum length. For example, in the StanWiFi dataset, sample lengths include 19,995, 19,980, 19,990, and so on. By setting the maximum length to 2000, the algorithm reduces all signal lengths to 2000 (Figure 6).

#### 3.3.4. Preprocessing: Feature Extraction

After the Wi-Fi signal is extracted using the sliding window method or through the time alignment technique, the Channel State Information (CSI) data are decomposed into amplitude and phase components as described in Equations (4) and (5).

#### 3.3.5. Normalization

CSI data collected from various environments or devices often exhibit variations in size. Normalization ensures that all data samples are standardized to a uniform size, to ensure compatibility with models requiring fixed input dimensions. In this context, normalization is applied to the amplitude across the datasets using the widely utilized min–max normalization technique for scaling data within a specific range. The formula for min–max normalization is shown in Equation (6).

(6) $$x^{\prime }=\frac{x-\min\left(x\right)}{\max\left(x\right)-\min\left(x\right)},$$

where *x* represents the original data point, min(*x*) is the minimum value in the dataset, max(*x*) is the maximum value in the dataset, and *x′* is the normalized value of *x*.

#### 3.3.6. Phase Unwrapping

The CSI phase provides critical information about wireless signal propagation, including path delays, multipath effects, and Doppler shifts. However, the phase information derived from CSI is often wrapped, meaning it is confined to a specific range, such as [−π, π] or [0, 2π]. This wrapping occurs because phase is typically expressed as an angle, and angles exceeding these bounds are wrapped back into the range.

**Figure 7:**
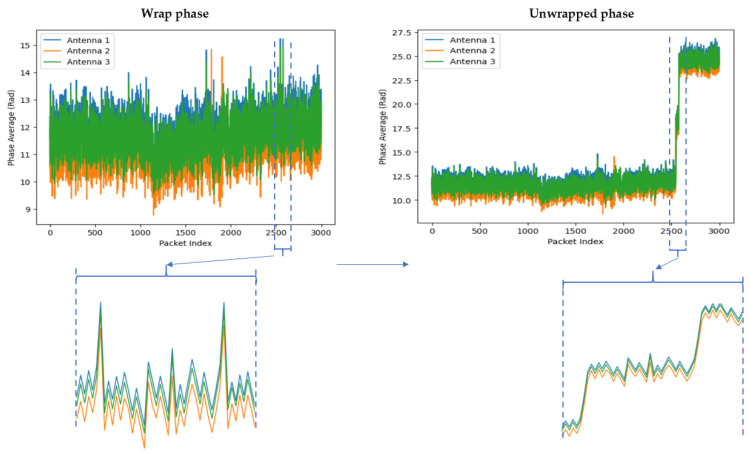
An example of phase unwrapping in CSI using for falling action.

Phase unwrapping is the process of resolving these phase ambiguities to create a continuous phase representation. This is achieved by identifying and correcting discontinuities introduced by wrapping, ensuring smooth phase progression over time or frequency. The visualization of phase unwrapping for falling action is illustrated in Figure 7, while the formula for phase unwrapping is provided in Equation (7).

(7) $${\hat{\Phi }}_{i}=\Phi_{i}+2\pi \frac{{\hat{\Phi }}_{i-1}-\Phi_{i}}{2\pi },$$

where ${\hat{\Phi }}_{i},\;\Phi_{i}$ are the unwrapped and raw phases at index *i*, respectively; ${\hat{\Phi }}_{i-1}$ is the previously unwrapped phase and the floor function ⌊⋅⌋ denotes rounding to the nearest integer.

#### 3.3.7. Gaussian Range Encoding

**Figure 8:**
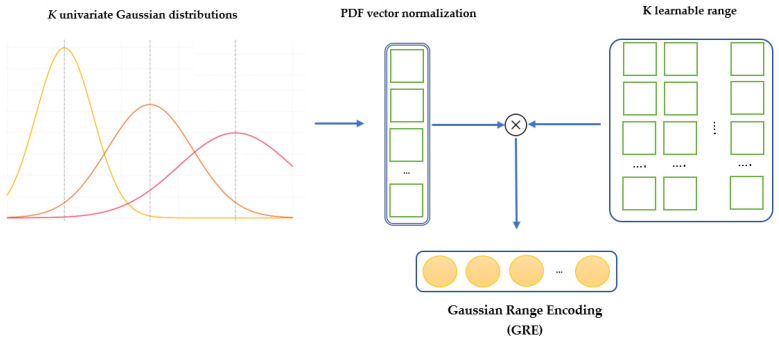
An example of GRE with K univariate Gaussian distributions.

Gaussian Range Encoding (GRE) is introduced in [32,37] as a mechanism to preserve order information from CSI data, which is crucial for recognizing sequential activities such as “sitting down” or “standing up”. Unlike traditional positional encoding methods, which assign unique encodings to individual time steps, GRE focuses on encoding ranges. This approach preserves temporal continuity and improves robustness against subtle variations in activity speeds or blank intervals within the sequence. The final representation of GRE is obtained by multiplying the *K*, learnable range of vector embeddings, with the probability density function (PDF) vector. The PDF is normalized across *K* Gaussian distributions. Figure 8 shows how data are mapped into Gaussian distributions and how these distributions are transformed into learnable embeddings for integration into the neural network.

#### 3.3.8. Multi-Scale Convolution Augmented Transformer (MCAT) Layer

**Figure 9:**
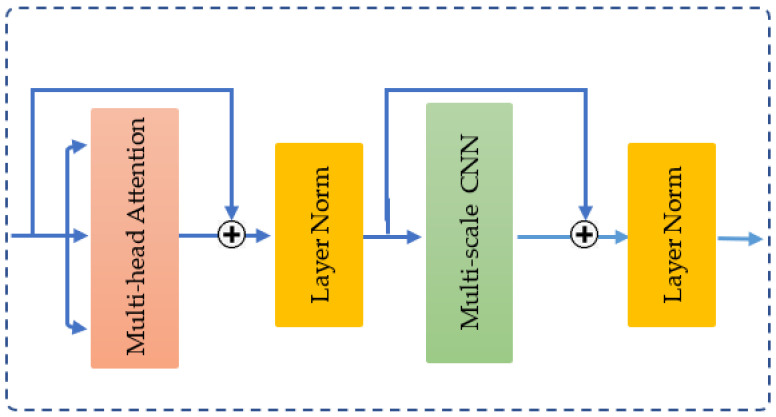
The structure of MCAT layer.

The MCAT layer [32] integrates two key components: a multi-head self-attention mechanism [29] and a multi-scale convolutional neural network. These components are connected using residual connections and layer normalization [38], as shown in Figure 9.

In the multi-head attention stage, the input $X\in \;{\mathbb{R}}^{L\times d_{in}}$, representing amplitude or phase—is transformed into three distinct vectors: query (*Q*), key (*K*), and value (*V*)—via three linear projections. Self-attention computes a weighted sum of the input values, where the weights are determined by the dot product between the query and the corresponding key. The process is defined mathematically as follows:

(8) $$Q=XW_{Q},\;\;\;W_{Q}\in \;{\mathbb{R}}^{d_{in}\times d_{k}}$$

(9) $$K=XW_{K},\;\;\;\;W_{K}\in \;{\mathbb{R}}^{d_{in}\times d_{k}}$$

(10) $$V=XW_{V},\;\;\;\;W_{v}\in \;{\mathbb{R}}^{d_{in}\times d_{v}}$$

(11) $$Attention\;\left(Q,K,V\right)=softmax\left(\frac{QK^{T}}{\sqrt{d_{k}}}\right)V,$$

here, $d_{k}$ represents the dimensionality of both the queries and keys, $\sqrt{d_{k}}$ is the scaling factor used to stabilize gradients and enhance computational efficiency.

To jointly attend to information from different representation subspaces, the vectors *Q*, *K*, and *V* are projected *h* times (referred to as *h-heads*) using distinct projection parameters. The outputs from these projections are then concatenated and passed through a final projection to generate the output:

(12) $$MultiHead\;\left(Q,K,V\right)=\left[head_{1};head_{2};\ldots ;head_{h}\right]W_{O},$$

where each $head_{i}=Attention\left(XW_{Q}^{i},\;XW_{K}^{i},XW_{V}^{i}\right)$ and $W_{O}\in {\mathbb{R}}^{hd_{v}\times d_{0}}$ is the final projection matrix.

Subsequently, the multi-scale CNN stage processes the output features *Y* from the multi-head self-attention module by applying learnable weights $W^{j_{i}}\in \;{\mathbb{R}}^{d_{0}\times j_{i}\times d_{0}}$. Here, *W^ij^* consists of *d_o_* filters, where each filter sized *j_i_* × *d_o_*. The output of the multi-cale CNN is presented as *P* = {*P*_1_,*P*_2_,…,*P_j_*}, where $P_{i}\in \;{\mathbb{R}}^{L\times d_{0}}$ is the feature matrix corresponding to the kernel size *j_i_* at index *i*. *P_i_* is computed as follows:

(13) $$P_{i}=ReLU(Dropout\left(BN\left(Conv\left(W^{j_{i}};Y\right)\right)\right),$$

Equation (12) involves a series of operations, including convolution, batch normalization (BN), dropout, and a rectified linear unit (ReLU) activation function.

#### 3.3.9. Gated Residual Network (GRN)

Gated Residual Networks (GRNs) [35] are a type of neural network architecture designed to improve information flow via residual connections while incorporating a gating mechanism to regulate the flow of features. The gating mechanism, typically implemented using a sigmoid function, selectively controls which parts of the input are passed through, allowing the network to prioritize the most relevant features. This combination of residual connections and gating mechanisms enables a GRN to more effectively capture complex patterns and improve model performance, especially in tasks that involve high-dimensional data or deep architectures. GRNs have been widely applied in various domains, including time series forecasting [36] and sequence modeling [39], which require learning long-term dependencies.

**Figure 10:**
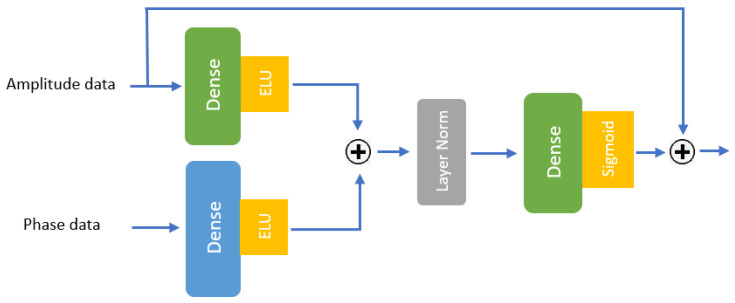
The structure of the GRN implemented in our model.

The structure of the GRN in our model is illustrated in Figure 10. This figure demonstrates how amplitude and phase data are processed through dense layers with exponential linear unit (ELU) activation functions, after which their outputs are summed using a residual connection (denoted by the summation symbol “+”). The resulting feature matrix undergoes layer normalization before being passed through another dense layer and a sigmoid gating mechanism. The final output is computed by integrating the gated outputs through an additional summation operation. The exponential linear unit (ELU) activation functions are defined as shown in Equation (14).

(14) $$ELU\left(x\right)=\left\{\begin{array}{cc} x, & if\;x>0\\ \alpha \left(\exp\left(x\right)-1\right), & and\;if\;x\leq 0 \end{array}\right.,$$

## 4. Experimental Evaluation

In this section, we present the experimental results for the three datasets introduced in Section 3.2 (MultiEnv, StanWiFi, and the MINE lab dataset). The evaluation metrics employed include accuracy (Acc), precision (Pre), recall, and F1-score. Additionally, we provide a comparative analysis of our results against the baseline and the state-of-the-art model.

### 4.1. Hyperprameters

In this subsection, we provide a concise summary of the symbols for the variables and hyperparameters utilized in the experiment, and their corresponding values, chosen to optimize performance (Table 3).

The StanWiFi and MultiEnv datasets were divided into training and validation sets using an 80:20 split ratio, with shuffling applied to ensure randomness. This approach aligns with common practices in the literature for utilizing the StanWiFi and MultiEnv datasets, as referenced in previous studies such as [16,22,24,26,27,31]. For the MINE lab dataset, a 5-fold cross-validation approach was employed to ensure robust evaluation, given its smaller size. The dataset was divided into five equal parts, with each fold serving as the test set once, while the remaining folds were used for training and validation.

The loss function deployed in this experiment is sparse categorical cross-entropy, which is defined in Equation (15):

(15) $$Loss\left(y,\hat{y}\right)=-\frac{1}{N}\overset{N}{\underset{i=1}{\sum }}\log\left({\hat{y}}_{i,y_{i}}\right),$$

where *N* represents the number of samples, *y_i_* is the true label, and ${\hat{y}}_{i,y_{i}}$ is the predicted probability for the true class *y_i_* for the *i-th* example.

### 4.2. Experimental Results on StanWiFi and MINE Lab Datasets

Table 4 compares models on the StanWiFi dataset based on accuracy, precision, recall, and F1-score. The models are listed chronologically, with the earliest model being THAT (2021) and CSITime (2022), both of which achieved over 98% accuracy but did not report precision, recall, or F1-score. Several other models, such as LiteHAR (2022) and CNN-GRU (2022), report high precision and recall, though not all models provide complete metric details. Notably, our model achieves the highest reported metrics: 99.93% accuracy, 99.86% precision, 99.95% recall, and 99.95% F1-score. This outperforms all other models in the table, demonstrating a significant improvement over previous works in the field.

Table 5 compares the performance of two models, THAT (2021), by Bing et al., and our model on the MINE lab dataset. The THAT (2021) model achieves consistent values across all metrics, scoring 97% for accuracy, precision, recall, and F1-score. Our model significantly outperforms the THAT model, achieving 99.24% across all evaluated metrics. This demonstrates a notable improvement on the MINE lab dataset, highlighting the superior capability of our model in accuracy, precision, recall, and F1-score. We selected the THAT model for comparison because it is publicly available online.

**Figure 11:**
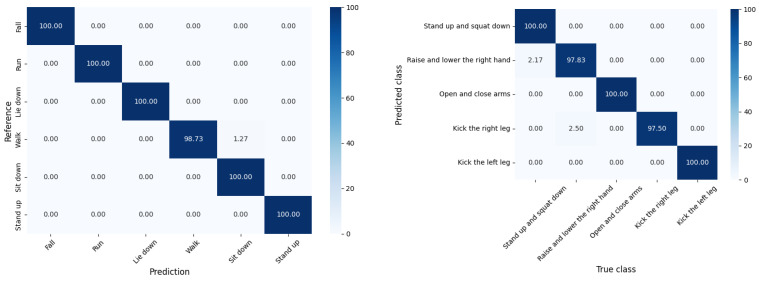
Model’s accuracy performance on StanWiFi datasets (**left**) and MINE lab dataset (**right**).

Figure 11 illustrates the model’s performance on six activities from the StanWiFi dataset on the left side: fall, run, lie down, walk, sit down, and stand up. The right side visualizes the model’s performance on additional activities: stand up and squat down, raise and lower the right hand, open and close arms, kick the right leg, and kick the left leg. The confusion matrices indicate that the model achieved a near-perfect classification for most activities, demonstrating high true positive rates across both datasets.

### 4.3. Experimental Results on MultiEnv Dataset

Table 6 summarizes the performance of various models on the MultiEnv dataset, divided into three different environments: E1: Office (LOS), E2: Hall (LOS), and E3: Room and hall (NLOS). In the E1 environment, various models such as SVM (2021, 2022), THAT (2021), STC-NLSTMNet (2023), and AAE+RF (2023) are compared. Our model achieves the highest accuracy (99.47%) and outperforms others in all metrics, including precision (99.48%), recall (99.47%), and F1-score (99.47%). In the E2 environment, comparisons include models such as SVM (2021, 2022), THAT (2021), and STC-NLSTMNet (2023). Again, our model achieves the best performance with an accuracy of 98.43%, precision of 98.01%, recall of 97.90%, and F1-score of 97.90%. In the E3 environment, our model once again excels with an accuracy of 98.78%, precision, recall, and F1-score all at 98.78%. This is superior to other models, such as THAT (2021), STC-NLSTMNet (2023), and AAE+RF (2023).

**Figure 12:**
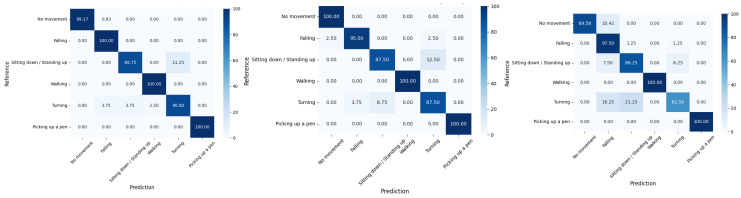
Confusion matrices for the MultiEnv datasets (E1: **left**; E2: **middle**; E3: **right**).

Figure 12 visualizes the model’s performance across six activities in the MultiEnv dataset running on E1, E2, and E3. These matrices show that the model achieved high accuracy across all activities.

## 5. Conclusions

In this study, we presented a novel approach for human activity recognition (HAR) using Wi-Fi sensing, leveraging both the phase and amplitude components of Channel State Information (CSI). Our proposed model, which combines attention-based mechanisms with a Gated Recurrent Network (GRN) architecture, outperformed state-of-the-art models in HAR. The model’s efficacy was rigorously tested across multiple datasets, including StanWiFi, MultiEnv, and the MINE lab dataset, showcasing its adaptability and robustness in both line-of-sight (LOS) and non-line-of-sight (NLOS) environments. The source code is available at https://github.com/thai-duy-quy/PA-CSI-HAR (access on 26 January 2025).

By utilizing both phase and amplitude signals, we addressed the limitations of prior models that predominantly focused on a single feature, leading to improved accuracy in recognizing complex human activities. Our experimental results indicate that the multi-feature approach of combining amplitude and phase information significantly enhances accuracy, precision, recall, and F1-score metrics compared to existing methods.

Despite these advancements, our method has certain limitations. Experiments conducted in controlled laboratory settings differ from real-world environments, where Wi-Fi signals are influenced by factors such as stationary and moving objects. Moreover, our current study does not include cross-environment testing, even though real-world Wi-Fi signals are significantly affected by the surrounding environment. In the future, we aim to address these challenges by developing solutions to improve the robustness and adaptability of our method for diverse and dynamic conditions.

## Figures and Tables

**Table 1:** Summary of public datasets.

Source	Dataset Name	Description
Alsaify et al. (2020) [15]	MultiEnv	This dataset was collected in three scenarios: line-of-sight (LOS) in both the office and hall, and non-line-of-sight (NLOS).
Yousefi et al. (2017) [16]	StanWiFi	This dataset contains continuous CSI data for six activities without precise segmentation timestamps for each sample.
Yang et al. (2022) [17]	Widar 3.0	This large dataset, collected using Intel 5300 NIC with 30 subcarriers and containing 258 K Wi-Fi-based hand gesture instances spanning 8620 min across 75 domains.
Guo et al. (2019) [18]	WiAR	This dataset includes 16 activities, comprising coarse-grained activities and gestures, performed 30 times each, by ten volunteers.
Yang et al. (2023) [19]	NTU-Fi	Collected using the Atheros CSI tool, this dataset features 114 subcarriers per antenna pair, and it includes 6 human activities and 14 gait patterns.
Meneghello et al. (2023) [20]	WiFi-80 MHz	Collected using two Netgear X4S AC2600 IEEE 802.11ac routers with 256 subcarriers (242 usable), this dataset features ten subjects and three applications

**Table 2:** Activities and class descriptions in the MultiEnv dataset.

Class	Activity	Description
0	No movement	Sitting, standing, or lying on the ground
1	Falling	Falling from a standing position or from a chair
2	Sitting down or standing up	Sitting down on a chair or standing up from a chair
3	Walking	Walking between the transmitter and receiver
4	Turning	Turning at the transmitter’s or receiver’s location
5	Picking up	Picking up an object such as a pen from the ground

**Table 3:** Hyperparameters and values chosen.

Hyperparameter	Values
Window size	StanWiFi dataset: 2000; our own dataset: 1000
Stride size	StanWiFi dataset: 200; our own dataset: 100
K-Gaussian encoding	10
Input data dimensions $(d_{in}\times d_{k}$)	StanWiFi dataset: (2000, 90); MultiEnv dataset: (850, 90); MINE lab dataset: (1000, 90)
Filter size in Multi-scale CNN (horizontal, vertical)	Horizontal: {10, 40}; Vertical: {2, 4}
Number of heads in the multi-head self-attention mechanism	h-head: 9; v-head: 50
Dropout rate	0.1
Number of dense layers in GRN	256
Optimizer	Adam (learning rate = 0.001; decay rate = 0.9)
Batch size	8
Epochs	200
Training environment	NVIDIA GeForce RTX 3060 with CUDA v. 12.4, Python 3.11, TensorFlow 2.16

**Table 4:** Results of the experiments on the StanWiFi dataset.

Source	Model	Acc	Pre	Recall	F1-Score
Li et al. [32]	THAT (2021)	98.20	-	-	-
Yadav et al. [24]	CSITime (2022)	98.00	-	-	-
Salehinejad et al. [26]	LiteHAR (2022)	98.00	99.16	98.87	99.01
Salaby et al. [27]	CNN-GRU (2022)	99.31	99.5	99.43	-
Islam et al. [28]	STC-NLSTMNet (2023)	99.88	99.72	99.73	-
Jannat et al. [5]	AAE+RF (2023)	99.84	99.82	99.83	99.81
Ours	PA-CSI (2024)	99.93	99.86	99.95	99.95

**Table 5:** The experiments on MINE lab dataset.

Source	Model	Acc	Pre	Recall	F1-Score
Li et al. [32]	THAT (2021)	97.00	97.00	97.00	97.00
Ours	PA-CSI (2024)	99.24	99.24	99.24	99.24

**Table 6:** The experiments on MultiEnv dataset.

Environment	Source	Model	Acc	Pre	Recall	F1-Score
E1: Office (LOS)	Alsaify et al. [15]	SVM (2020)	94.03	-	-	-
	Li et al. [32]	THAT (2021)	98.95	98.28	98.26	98.26
	Alsaify et al. [22]	SVM (2022)	91.27	-	-	-
	Islam et al. [28]	STC-NLSTMNet (2023)	98.20	98.10	98.08	98.09
	Jannat et al. [5]	AAE+RF (2023)	97.65	96.42	96.41	94.40
	Ours	PA-CSI (2024)	99.47	99.48	99.47	99.47
E2: Hall (LOS)	Alsaify et al. [15]	SVM (2020)	94.03	-	-	-
	Li et al. [32]	THAT (2021)	97.39	97.24	97.22	97.22
	Alsaify et al. [22]	SVM (2022)	91.27	-	-	-
	Islam et al. [28]	STC-NLSTMNet (2023)	96.65	96.54	96.41	96.48
	Ours	PA-CSI (2024)	98.43	98.01	97.90	97.90
E3: Room and hall (NLOS)	Li et al. [32]	THAT (2021)	97.56	97.04	97.04	97.03
	Islam et al. [28]	STC-NLSTMNet (2023)	94.68	94.57	94.55	94.56
	Jannat et al. [5]	AAE+RF (2023)	93.33	93.12	93.07	93.14
	Ours	PA-CSI (2024)	98.78	98.79	98.78	98.78

## Data Availability

The StanWiFi dataset is available at https://github.com/ermongroup/Wifi_Activity_Recognition (accessed on 6 February 2025). The MultiEnv dataset is available at https://data.mendeley.com/datasets/v38wjmz6f6/1 (accessed on 6 February 2025). The MINE lab dataset is provided upon request by contacting the corresponding author due to author privacy concerns.
